# 4078 Identification of distinct fibroblast populations with unique roles in pancreatic cancer progression and tumor immunity

**DOI:** 10.1017/cts.2020.301

**Published:** 2020-07-29

**Authors:** Josephine Kebbeh Darpolor, Xiaofeng Zheng, Sujuan Yang, Hikaru Sugimoto, Julienne Carstens, Pedro Correa de Sampiao, Valerie LeBleu, Raghu Kalluri

**Affiliations:** 1 The University of Texas Health Science Center at Houston

## Abstract

OBJECTIVES/GOALS: The desmoplastic reaction in PDAC involves a significant accumulation of immune cells and fibroblasts.The functional diversity of carcinoma associated fibroblasts (CAFs) remains largely unknown, and identification of immune regulating subsets would have a substantial impact in augmentation of immunotherapy efficacy. METHODS/STUDY POPULATION: Employing histology, FACs, multiplex immunohistochemistry, single cell RNA sequencing (sc-RNA-seq) and genetically engineered mouse models, we demonstrate that aSMA^+^ cells are a dominant CAF population in PDAC with tumor restraining properties (TS-CAFs), as opposed those of the FAP^+^ CAFs, which demonstrate tumor promoting activity (TP-CAFs). RESULTS/ANTICIPATED RESULTS: Analysis of bulk tumor depleted of either TS-CAFs or TP-CAFs showed that TS-CAFs predominantly modulate extracellular matrix (ECM) production, facilitate cell-ECM adhesion and regulate adaptive immunity, while TP-CAFs exhibit a lineage that is skewed towards a pro-inflammatory, chemokine secreting phenotype. Further, scRNA-Seq analyses demonstrate that CAFs share distinct gene expression profiles characteristic of lymphocytic and myeloid lineages. Together our data distinguish two populations of CAFs, one which is tumor suppressing with roles in ECM remodeling and another which is tumor promoting with roles in cytokine production, both with immune modulating capabilities. DISCUSSION/SIGNIFICANCE OF IMPACT: Our study identifies a complex network of functionally heterogeneous fibroblasts during PDAC progression with significant immunotherapeutic implication. The identification of distinct fibroblast subsets will allow us to discriminately target fibroblast populations to augment immunotherapy efficacy in pancreatic cancer.

